# SARS-CoV-2 active infection and antibodies amongst health personnel during the outbreak in Cameroon: Strengthening the health system for response to future public health emergencies

**DOI:** 10.1371/journal.pone.0304477

**Published:** 2024-05-31

**Authors:** Sen Claudine Henriette Ngomtcho, Blaise Mboringong Akenji, Ketina Hirma Tchio-Nighie, Joseph Fokam, Etienne Guenou, Carolle Nsa’Amang Eyebe, Yvan Junior Nzegni Kamkoum, Valdex Derick Ntale Tchoffo, Collins Buh Nkum, Hervé Christian Tchoudjin Paho, Yvette Marie Solange Ebogo, Aude Nanfak, Martin Maidadi-Foudi, Crescence Fouda, Angyiba Serge Andigema, Lilian Nsah Bongdze-em, Beri Nadin Nfor, Judith Torimiro, Anne Cécile Zoung-Kanyi Bissek, Michel Noubom, Marie Claire Assoumou Okomo, Jérôme Ateudjieu

**Affiliations:** 1 National Public Health Laboratory, Ministry of Public Health, Yaoundé, Cameroon; 2 Department of Microbiology, Hematology and Immunology, Faculty of Medicine and Pharmaceutical Sciences (FMPS), University of Dschang, Dschang, Cameroon; 3 Genomic Surveillance Study Group, Ministry of Public Health, Yaoundé, Cameroon; 4 M.A. SANTE (Meilleur Accès aux Soins de Santé), Yaoundé, Cameroon; 5 Chantal Biya International Reference Centre for Research on HIV/AIDS Prevention and Management (CIRCB), Yaoundé, Cameroon; 6 Department of Biological Sciences, Faculty of Medicine and Pharmaceutical Sciences (FMPS), University of Douala, Douala, Cameroon; 7 Centre de Recherche en Maladies Emergentes et Re-emergentes (CREMER), Yaoundé, Cameroun; 8 Division for Operational Health Research (DROS), Ministry of Public Health, Yaoundé, Cameroon; 9 Department of internal Medicine, Faculty of Medicine and Biomedical Sciences, University of Yaoundé I, Yaoundé, Cameroon; 10 Department of Public Health, Faculty of Medicine and Pharmaceutical Sciences, University of Dschang, Dschang, Cameroon; University of Sharjah, UNITED ARAB EMIRATES

## Abstract

**Background:**

Health personnel (HP) are on the frontlines during response to public health emergencies like COVID-19. This risk of exposure suggests the need for safety in responding to any pandemic. Therefore, to ascertain the rate of SARS-CoV-2 infection and immunity, and their determinants amongst HP become relevant.

**Methods:**

A cross sectional health facility-based study was carried-out amongst HP in the Centre Region of Cameroon from 1^st^ February to 30^th^ June 2021. Characteristics and access to preventive tools were collected using face-to-face administered questionnaire. Nasopharyngeal swabs and whole blood were collected for PCR, IgG and IgM testing respectively. STATA version 17 software was used for data analysis. Determinants of COVID-19 infection were explored by estimating crude and adjusted Odd Ratio.

**Results:**

Out of 510 HP reached, 458 were enrolled with mean age of 35 (±10) years. Thirty-four (7.4%) were PCR-positive to SARS-CoV-2 with 73.5% being clinicians versus 9 (26.4%) non-clinicians (p = 0.05). Sero-positivity to SARS-CoV-2 IgG/IgM was 40.2% (184/458), with 84.2% being clinicians versus 29 (15.8%) non-clinicians (p = 0.733). Amongst the 34 HP with PCR-positivity, 16 (47%) had no antibodies, while, 15 (44%) were IgG only. An estimate of HP (43.7%) had at least an evidence of PCR, IgG or IgM contact to COVID-19. Determinants of PCR-positivity was being clinical staff (AOR = 0.29, P = 0.039); and that of IgG/IgM were being non clinical staff (AOR = 0.41, p = 0.018) and regular use of face masks (AOR = 0.44, p = 0.001). HP trained on IPC (24%) were mainly from peripheral level (74.7%, p = 0.002).

**Conclusion:**

Active infections were within the range of pandemic control (<10%). However, around two-fifths of participants have had contact with the virus, indicating that HP remains a population at risk of COVID-19 and other similarly-transmitted epidemic prone diseases, and also an important source of transmission. There is need of vaccine to achieve protectiveness, and optimal response also requires capacity building to improve the health system when challenged by a future pandemic.

## Introduction

COVID-19 (coronavirus disease 2019), with the causative agent SARS-CoV-2 (Severe Acute Respiratory Syndrome Coronavirus 2) poses enormous challenges to healthcare systems. Its response involves the participation of various parties with health facilities having the mission to identify and manage cases amongst others. Health personnel (HP) are on the frontlines of this global crisis with the substantial task to diagnose, care and treat an exponentially growing number of extremely and critically ill patients [1, 2]. They may experience an increased risk of SARS-CoV-2 infection due to their close contact with highly infectious patients, but also due to exposure to undiagnosed or subclinical infectious cases. Infection of HP can have significant consequences both on an individual and at the community level, as it may serve as a potential source of transmission within the healthcare environment and to the general population [3]. Based on studies conducted in Hong Kong, Japan, Singapore, Taiwan, Thailand, and Vietnam on COVID-19 infection amongst all essential workers, health professionals were the most affected workers [4]. At the end of the first wave, Cameroon was faced with an increase in the number of COVID-19 cases. 30,740 cumulative cases and 474 deaths were confirmed as of 31 January 2021 with a case fatality rate of 1.6 and a bed occupancy of 3.6%. At the beginning of the second wave, started on the African continent on January, 27^th^ 2021, the Public Health Emergency Operations Coordination Center (PHEOCC), in Cameroon, reported more than 1.400 active cases including 113 hospitalized with 22 receiving respiratory assistance. The overall case fatality rate for COVID-19 in Cameroon was 2%, and the case fatality rate amongst health professionals 2.4% [5]. This was a call for concern for high risk groups, such as HP, in the context where African Nations Championship (CHAN 2021) which began on 16 January 2021 and the prominence of a more contagious strain of the virus, the Omicron strain needed more commitment from HP, hence their higher level of exposure as compared to the general population. Dzinamarira *et al*. [6] reported that health care workers are more than ten times more likely to be infected with Coronavirus Diseases 2019 than the general population, thus demonstrating the burden of COVID-19 amongst them [7, 8].

Exposure can be ascertained through detectable antibodies against SARS-CoV-2 [9, 10]. A high proportion of the SARS-CoV-2 infections in health care workers have been notified worldwide and antibody seroprevalence has been shown to be higher in that group than in the general population [11]. Such antibody response has been shown to correlate with protective immunity against infection [12, 13]. The exposure of HP could reduce the workforce availability and hinder an effective response in case of emergency [14, 15]. Therefore, it is relevant to evaluate the burden of SARS-CoV-2 and its potential determinants amongst HP in COVID-19 management facilities in Cameroon. We determined here the proportion of COVID-19 active cases and the degree of exposure to SARS-CoV-2 amongst HP. With the dispersion of cholera in the country, public health preparedness must be extended to non-endemic areas. Human resources, testing capacities, and personal protective equipments (PPEs) are needed for quick interventions and emergency responses. Thus we also assessed preventive measures and capacity building in health facilities to respond to future epidemics beyond COVID-19.

## Materials and methods

### Study design

This was a cross sectional and facility-based study carried-out from 1^st^ February to 30^th^ June 2021 amongst HP in 7 health facilities of the Centre region of Cameroon, selected on the base of their involvement in the response to the crisis with the task to manage, diagnose or treat patients. It targeted health professional involved directly or indirectly in the management of COVID-19 cases, recruited using a convenient sampling technique. Data on participants’ characteristics activities, access to preventive tools and capacity building were collected using a face-to-face administered questionnaire. Additionally, PCR was performed on nasopharyngeal swabs to identify COVID-19 active cases, and antigen RDTs used on blood samples to detect IgG and IgM antibodies against SARS-CoV-2.

### Study population

The study targeted health professionals of the Centre region providing care to COVID-19 patients or working where such patients were quarantined. In the context of the present study, HP are defined as “all staff involved in the provision of care to a COVID-19 patient” and further include allied and auxiliary health workers such as cleaning and laundry personnel, X-ray physicians and technicians, clerks (including admission/reception clerks), phlebotomists, respiratory therapists, social workers, physical therapists, laboratory personnel, managers.

### Data collection tools and procedures

Participants were face to face interviewed using a questionnaire designed by the research team. Sociodemographic data on participants, the number of year of professional experience, the availability of PPE for the personnel in health facilities, training of health personnel on IPC (Infection Prevention Control) and the use of barrier measures were the main variables collected. The main activities were giving care to COVID-19 patients and testing of suspected cases.

### Samples collection and processing

Nasopharyngeal swabs were collected following the standardized procedures defined by the WHO [16]. Two sterile swabs were used; the first one was inserted into the right nostril and introduced into the viral transport medium for PCR testing and the second one in the left nostril and introduced into the buffer for antigen RDT. Venous blood sample was collected from the vein in the anterior region of the elbow directly into vacutainer dry tubes (Sarstedt, Nümbrecht, Germany) and allowed to stay on the bench for at least two hours. All samples were transported and processed at the Cameroon National Public Health Laboratory (NPHL). Once in the laboratory, each blood sample was centrifuged at 3,000x *rpm* for 15 min, and the serum sample aliquoted into a 1.5 mL cryotube for anti SARS-CoV-2 IgG/IgM antibodies detection using the rapid immunochromatographic colloidal gold-conjugate test (Panbio, Abbott, California USA) following manufacturer’s recommendations.

Regarding PCR, nasopharyngeal swabs were inactivated by heat for 15 minutes at 72°C using a digital block heater (VWR, Troemner, USA). Viral RNA extraction and purification was performed manually by spin-column technique using the QIAamp RNA Mini kit (Qiagen Str, Hilden, Germany). The purified RNA was amplified in an ABI 7500 Thermofisher thermal cycler using a one-step RNA PCR-Taqman fluorescence Probe kit from Daa n Gene Co, Ltd of Sun Yat-sen University. This kit targets and detects two SARS-CoV-2 genes, the N gene and the ORF1ab gene [17] via the FAM (N gene) and VIC (ORF1ab gene) channels, while detecting the human RNase P gene via the CY5 channel (internal control). Samples with Ct (Cycle threshold) values below 38 for both genes were considered positive. In the case only one gene had a CT < 38, PCR was repeated and, if a single gene or both returned Ct values <38, the sample was considered positive. Samples with no detection of internal control were also re-tested.

### Statistical analysis

Data collected (S1 Text) was analyzed using STATA version 17 software. Qualitative variables were analyzed as proportions and quantitative variables as mean and standard deviation. The analysis of associations was carried out using the crude Odds Ratio, the chi-square test and logistic regression. Bivariate analysis was carried out using the Chi2 test and crude Odds Ratio. Regression was done to calculate the adjusted Odds Ratio by including in the model all variables that were associated during the bivariate analysis. The significance threshold was set at 5% (p< 0.05).

### Ethical consideration

All participants were fully informed about the study and only those that provided written consent were recruited. To avoid the risk to contaminate participants, all the members of the study team wore face masks during the whole recruitment procedure. To reduce pain or discomfort during nasopharyngeal swab or blood collection, sample were collected by experienced healthcare workers. The protocol was approved by the Cameroon National Ethical Committee for Research on Human Health (reference 2020/05/1223/CE/CNERSH/SP).

## Results

A total of 510 HP were reached, out of which 458 consented to participate in the study, giving a response rate of 89.8%. The sex ratio was 0.73 in favor of females. The average age was 35±10 years old.

### Characteristics of health structures and health personnel recruited

**Fig 1:**
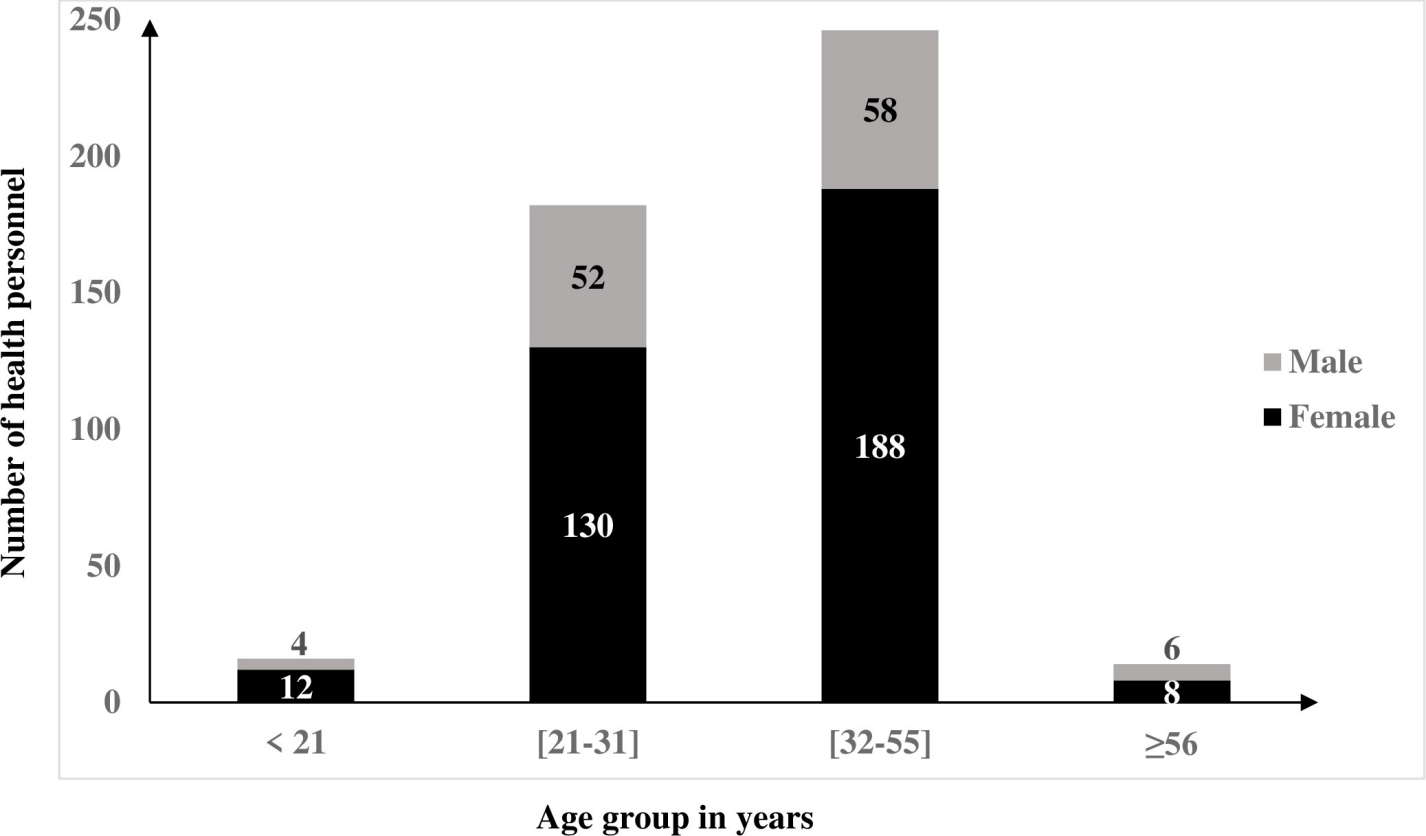
Gender and age distribution of health personnel recruited in the study.

**Fig 2:**
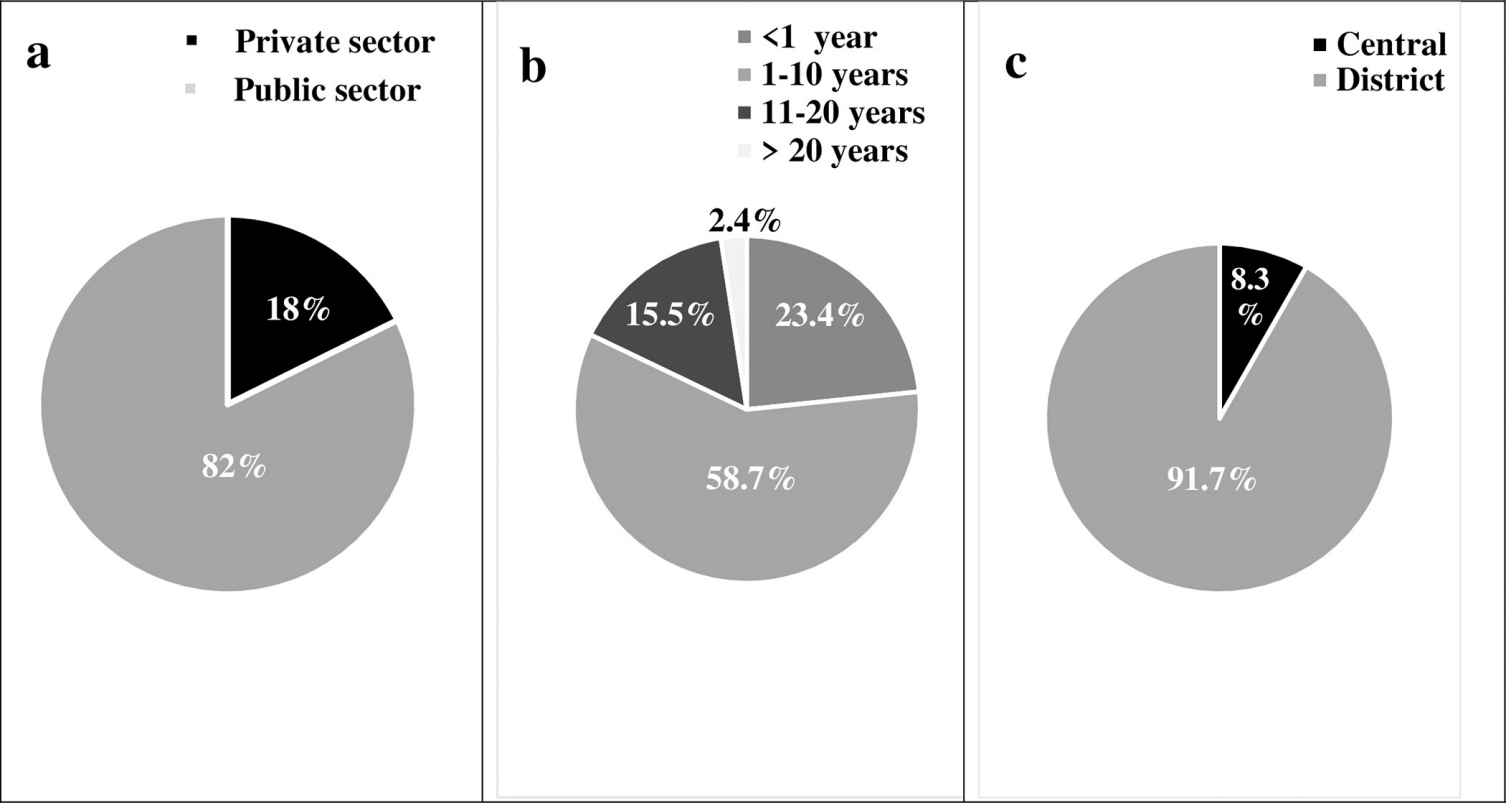
Health sectors, work experience and level of health facilities of HP. (a): Sectors to which selected health facilities belong. (b): number of years of work experience of HP. (c): Level on the health pyramid to which selected health facilities belong.

Of the 458 respondents, 69 (15%) were non clinical staff (NCS) while 389 (85%) were clinical staff (CS). Amongst CS recruited 167 (36.46%) were nurses; 48 (10.5%) medical doctors and 79 (17.24%) laboratory personnel. The distribution of health personnel according to gender and age groups are presented in Fig 1. That of work experience; health facilities according to type of sector and level on the health pyramid are presented in Fig 2. About 4/5 (377/458) of HP worked in the public sector and 1/4 (107/458) had less than a year of professional experience.

### Prevalence of SARS-CoV-2 amongst health professionals

Less than 10% of HP were infected with SARS-CoV-2. CS were the most affected. Those with less than 1 year of professional experience are more likely not to contract the disease (Odd = 0.29, P = 0.049, IC = 0 .09–0 .99) (Table 1).

**Table 1:** Prevalence of SARS-CoV-2 amongst health professionals with regards to the category of staff and number of year of experience.

Variables	Modalities	Infection with SARS-CoV-2		Total (%)	X^2^	OR (IC)	P
		Infected (%)	Not infected (%)				
**Category of staff**	CS	25 (73.5)	364 (67.7)	389 (85.0)	3.73	0.46 (0.20–1.03)	0.053
	NCS	9 (26.5)	60 (6.8)	69 (15.0)			
**Total**		34 (100.0)	424 (100.0)	458 (100.0)			
**Work Experience (year)**	<1	3(8.8)	104(24.5)	107(23.4)	4.5	0.29 (0.09–0.99)	0.049
	1–10	23(67.6)	246(58.0)	269 (58.73)			
	11–20	7(20.6)	64(15.1)	71(15.5)			
	≥21	1 (2.9)	10(2.4)	11(2.4)			
** Total**		34(100.0)	424(100.0)	458(100.0)			

^CS^Clinical Staff

^NCS^Non Clinical Staff

### Seropositivity to SARS-CoV-2 IgG/IgM antibodies

40.2% (184/458) of HP developed antibodies against SARS-CoV-2. Of these, 84.2% (155/184) were CS versus 15.8% (29/184) NCS, p = 0.733 (Table 2). Those using masks regularly were found to have anti-SARS-CoV-2 antibodies the most (Table 3).

**Table 2:** Seropositivity to SARS-CoV-2 IgG/IgM antibodies with respect to family of activity.

IgG/IgM antibodies	Category of staff		Total (%)	X^2^	P
	NCS (%)	CS(%)			
Positive	29 (15.8)	155(84.2)	184 (40.2)	0.116	0.733
Negative	40 (14.6)	234 (85.4)	274 (59.8)		
Total	69 (15)	389(85)	458 (100)		

^CS^Clinical Staff

^NCS^Non Clinical Staff

**Table 3:** Seropositivity to SARS-CoV-2 IgG/IgM antibodies with respect to health departments and use of face masks.

		IgG/IgM antibodies		X^2^	P
		Negative	Positive		
**Health departments**	Emergency	29(10.6)	20(10.9)	22.9	0.018
	Laboratory	59(921.5)	45(24.5)		
	Internal medicine	47(17.2)	30(16.3)		
	Pediatry	12(4.4)	12(6.5)		
	Pharmacy	7(2.6)	9(4.9)		
	Surgery	12(4.4)	16(8.7)		
	AIDS care unit	49(17.9)	18(9.8)		
	Gynecology-Maternity	25(9.1)	13(7.1)		
	Cardiology/diabetology	6(2.2)	1(0.5)		
	Hygiene	7(2.6)	0(0.0)		
	Administration	7(2.6)	12(6.5)		
	Others	14(5.1)	8(4.3)		
**Use of face masks**	Always	169(61.7)	138(75.0)	8.84	0.03
	Sometimes	105(38.3)	46(25.0)		

### Association between seropositivity to IgG/IgM anti SARS-CoV-2 antibodies and PCR-positivity amongst HP

Acute and chronic seropositivity to SARS-CoV-2 IgM and IgG antibodies respectively was evaluated with active infections amongst HP. Amongst the 34 HP with PCR-positivity, while one half had no antibodies, the other half was dominated with IgG (15/34) antibodies. Specifically, IgG-alone was 70.6% (130/184) versus 16.3% (30/184) for IgM-alone (Table 4).

**Table 4:** Association between seropositivity to IgG/IgM anti SARS-CoV-2 antibodies and PCR-positivity amongst HP.

PCR	Anti-SARS-CoV-2 detected (%)			Anti-SARS-CoV-2 not detected	Total (%)	x^2^	p
	IgM&IgG	IgM	IgG				
**Positive**	2(5.8)	1(2.9)	15(44)	16 (47)	34	4.96	0.17
**Negative**	22	29	115	258	424		
**Total**	24	30	130	274	458		

### Determinants of PCR-positivity and anti SARS-CoV-2 exposure

#### Determinants of PCR-positivity

Determinants of PCR-positivity was being clinical staff (AOR = 0.29, P = 0.039) (Table 5).

**Table 5:** Determinants of PCR-positivity.

	Modalities	OR	SE	p	IC95
**PPE**	Mask sometimes	0.85	0.39	0.726	0.34–2.10
**Category of staff**	Clinical staff	0.29	0.17	0.039	0.09–0.93
**Health departments**	Laboratory	0.56	0.39	0.412	0.14–2.23
	Internal medicine	0.69	0.49	0.610	0.17–2.82
	Pediatric	0.49	0.57	0.541	0.05–4.76
	Pharmacy	1.21	1.14	0.836	0.19–7.66
	Surgery	0.73	0.67	0.739	0.12–4.44
	AIDS care unit	0.33	0.28	0.195	0.06–1.74
	Gynecology/Maternity	1.82	1.33	0.414	0.43–7.68
	Cardiology/Diabetology	1.34	1.78	0.823	0.10–17.7
	Hygiene	0.42	0.58	0.553	0.02–6.14
	Administration	0.53	0.56	0.549	0.06–4.23
	Others	0.71	0.67	0.725	0.11–4.54
**Work experience (years)**	1–10	3.26	2.09	0.064	0.93–11.44
	11–20	3.30	2.40	0.100	0.79–13.74
	≥21	1.88	2.50	0.635	0.13–25.48

#### Determinants of anti SARS-CoV-2 exposure

Determinants of antibodies exposure were being clinical staff (AOR = 0.41, p = 0.018) and regular use of face masks (AOR = 0.44, p = 0.001) (Table 6).

**Table 6:** Determinants of anti SARS-CoV-2 antibodies production.

	Anti SARS-CoV-2 Detection	OR	SE	P	IC95
PPE	Masks always	0.62	0.21	0.003	0.20–1.03
Category of staff	Clinical staff	0.41	0.15	0.018	0.20–0.86
Health departments	Laboratory	1.27	0.46	0.412	0.62–2.59
	Internal medicine	0.76	0.29	0.610	0.36–1.62
	Pediatric	1.29	0.65	0.541	0.47–3.49
	Pharmacy	1.58	0.94	0.836	0.49–5.07
	Surgery	1.71	0.83	0.739	0.65–4.47
	AIDS care unit	0.44	0.19	0.195	0.18–1.07
	Gynecology/Maternity	0.67	0.30	0.414	0.27–1.65
	Cardiology/Diabetology	0.30	0.35	0.823	0.03–2.94
	Administration	1.52	0.98	0.549	0.43–5.39
	Others	0.65	0.35	0.725	0.22–1.90
Work experience (years)	1–10	1.28	0.31	0.064	0.78–2.09
	11–20	1.20	0.40	0.100	0.62–2.32
	≥21	0.91	0.67	0.635	0.21–3.88

### Capacity building of health professionals based on their training on IPC and PPE availability

Almost ¼ (24%) of HP were trained on IPC with higher (76.4%) belonging to health facilities from public sector compared to those from private sector (p = 0.06); and working in a health facility at the central level on the health pyramid gave thrice more chance to be trained compared to that at peripheral level (AOR = 2.84, p = 0.002) (Table 7).

**Table 7:** Capacity building of health professionals based on their training on IPC and PPE availability.

Modalities		Training on IPC		X^2^	P	AOR	Availability of PPE		X^2^	P	AOR
		No	Yes				No	Yes			
**Level of health facility on the health pyramid**	Central	21	17(25.3)	9.74	0.002	2.84	0	38(25.3)	85.08	0.000	-
	Peripheral	327	93(74.7)				308	112(74.7)			
	Total	348	110 (24.0)				308	150			
**Health sector**	Private	55	26(23.6)	3.52	0.061	0.60	39	42(28.0)	16.3	0.000	2.68(1.6–4.3)
	Public	293	84(76.4)				269	108 (72.0)			
	Total	348	110 (24.0)				308	150			
**Health departments**	Emergency	40	9(8.2)	68.2	0.007		36	13(8.7)	155.3	0.000	3.2(2.0–5.0)
	Hygiene	05	2(1.8)				7	0(0.0)			
	Laboratory	79	25(22.7)				48	56(37.3)			
	Internal medicine	73	4(3.6)				76	1(0.7)			
	Administration	16	3(2.7)				4	15(10.0)			
	Cardiology/diabetology	03	4(3.6)				1	6(4.0)			
	Gynecology/maternity	32	6(5.5)				37	1(0.7)			
	Pediatric	21	3(2.7)				22	2(1.3)			
	Pharmacy	07	9(8.2)				2	14(9.3)			
	Surgery	21	7(6.4)				23	5(3.3)			
	AIDS care unit	31	36(32.7)				31	36(24.0)			
	Others	20	2 (1.8)				21	1(0.7)			

### Occurrence of contact cases with SARS-CoV-2 amongst health personnel

Contact of health personnel with SARS-CoV-2 was ascertain with a positive reaction obtained either with PCR or rapid IgM or IgG antigen testing. This study revealed that almost half (43.7%) of HP have had contact with the virus (Table 8).

**Table 8:** Occurrence of contact cases with SARS-CoV-2 amongst health personnel.

	PCR	IgM	IgG	IgM or PCR	IgM or IgG or PCR
**Positive cases**	34	54	154	85	200
**Total**	458	458	458	458	458
**Prevalence (%)**	7.4	11.8	33.6	18.6	43.7

## Discussion

This study evaluated the burden of SARS-CoV-2 and its potential determinants amongst health personnel (HP) from health facilities of the Centre Region of Cameroon during the second wave of the epidemic, with the aim to respond to future epidemics beyond COVID-19.

The highest proportions of patient’s diseases during the first, second, third and fourth waves of the epidemics in Cameroon were 2.7%, 2.0%, 15% and 1.1% respectively in the general population [18]. Here we report an overall proportion of 7.4% of active SARS-CoV-2 infection amongst HP. This shows an increase of COVID-19 active infections amongst HP during the second wave compared to the general population. This observation could be due to the fact that health personnel are at a higher risk of infection in a pandemic setting, based on their dual exposure in the community and in health facilities. Though the occurrence of active cases was higher amongst CS than NCS (p = 0.05), that prevalence was within the range of pandemic control (3%-19%) [19–23]; indicating a good response of the health system in terms of protecting those at the frontlines.

The overall sero-positivity to SARS-CoV-2 IgG and IgM antibodies was 41.2%, which did appear to be higher, compared to only 11% reported in 2021 based on a pooled data [24]. The risk of exposure was less amongst CS (OR 0.4; 95% CI 0.19–0.8) compared to NCS. This result is supported with that of Alishaq *et al*. (2021) [25] for who NCS (OR 1.21; 95% CI 1.09,1.34) was associated with a higher risk of infection which could be due to poor infection control. It is likely that CS are well trained on how to use barrier measures to prevent contracting the virus compared to NCS. Moreover, during the first wave of the epidemic, WHO promoted a staff contingency plan as pivotal to prevent the health care system from collapsing during a crisis such as the covid-19 pandemic. Therefore, each health facility had to take measures to prevent staff shortages. NCS might have been reallocated to clinical practices to ensure hospital staff capacity during that period. Then we will have less exposed CS to the disease. As observed with active cases, it was higher amongst CS (84.2%) than NCS (15.8%), but the difference was not significant (p = 0.733). Korth et al. [26]; Fusco et al. [27] and Alishaq et al. [25] found a different pattern between these two groups of staffs. While the lower risk of contracting the virus amongst CS could be attributed to increased awareness on COVID-19 and infection prevention measures they adopt in response to the epidemic. The misuse of barrier measures with those in direct contact with infected patient might explain high exposure of CS compared to NCS in our study; This could have led to a silent spread of the virus thus the importance of continuous training. In a systematic review [28], the overall seroprevalence of SARS-CoV-2 antibodies was 8.7%. A National seroprevalence survey in Cameroon [29] reported 10.5% amongst which 46.3% tested IgG and IgM positive, 45.8% tested IgG positive and 7.9% tested only IgM positive. This seroprevalence was highest in the Centre Region (15.4%) where this study was undertaken. The same pattern was observed with IgG and IgM in the community: 45.8% and 7.9% [18] and amongst healthcare workers (44.7 et 6.5) [30]. In our health facility- based study, we reported even higher IgG and IgM (70.6% and 16.3% respectively). This shows the ongoing spread of the virus not only in the community, but also in health facilities over time. Moreover, we looked at the occurrence of contact cases with SARS-CoV-2 amongst recruited participants. Our results revealed that almost half of health personnel were in contact with the virus. This increase of level of exposure might be allocated to seropositivity acquired through vaccination. However, this hypothesis should be raised with some restriction, given that COVID-19 vaccination campaign, started on April 26, 2021, was surrounded with vaccine hesitancy and, only 5.4% of the target population (not exclusive to health personnel) had received a single dose at the end of the campaign on April 30, 2021 [31]. Exposure to COVID-19 therefore still remains a matter of concern for this specific group of population.

The top three departments most exposed and infected were those of laboratory, internal medicine and emergency with 24.5%, 16.3% and 10.9% respectively; while some studies in Asia [24] and in America [31] rather reported medicine and emergency; and surgery and pediatric units respectively as having a significantly higher percentage of SARS-CoV-2 antibodies. This could be attributed to the fact that those health departments directly interact with and treat COVID-19 patients, and more often in Emergency, can be found patients with unknown COVID-19 infectivity status. However, the significantly higher (p = 0.018) percentage of SARS-CoV-2 antibodies amongst laboratory personnel reported here might be explained by their involvement in the screening of already suspected samples which are potentially infectious and are therefore increasing the risk of exposure. This could be also the reason why they were giving the priority with PPE. In fact, we found that there was twice more chance to be provided with PPE while working in the laboratory compared to other departments (OR 3.2; 95% CI 2.0–5.0, p = 0.000).

Thought in some studies people with anti SARS-CoV-2 were all PCR negative, it is not clear whether previous exposure to the virus prevents an acute infection. Almost 50% of HP with SARS-CoV-2 active cases in this study, were found IgG antibodies reactive, meaning they were previously exposed (Table 4). The occurrence of antibodies does not therefore guarantee immunity, whether be protection from SARS-CoV-2 infection nor does the absence of antibody reliably indicate susceptibility to infection or disease. Our results are in agreement with that of Alishaq et al. [32] who reported even higher (81%) antibodies positivity amongst PCR positive HP. This suggest that first exposure may therefore not been protective to a new infection as it was previously thought [33].

So as to avoid human resources shortage, hospital management needed, thought it was critical, to nominate HP who are at the lowest infection risk to be mobilised, such as those who are younger, basically the less experienced personnel (23.4%). This corroborates with the proportion of COVID-19 active cases which was lower in this group of experienced HP (OR = 0.29; 95%CI: 0.089–0.99; P = 0.049) (Table 1).

At the beginning of the epidemic, Public health systems in many countries were not adequately prepared to deal with the sudden surges in demand for human resources and infrastructures. Hospital transformation to COVID-19 treatment centers, started with Djoungolo Hospital and the creation of special centres for COVID-19 care such as ORCA; and a PHEOCC were necessary due to an increase in COVID-19 cases in the State. However, the functionality of these centres in health emergencies settings like the COVID-19 pandemic is still discussed. Despite the importance of such structures, they still face issues in overall coordination in the health sector, governance, infrastructure, planning and data flow [18]. A low compliance with PPE precautions was observed [34] worldwide, and there was an increase in PPE demands. And when it was available, their adequacy were still another issue [35, 36]. This observation agrees with our results where there was a significant difference (P = 0.000) on the availability of PPE at the health departments, health structures and category. Less than half of HP had PPE available during the second wave of the epidemic. Being in Departments involved in screening of pathogen agents such as laboratories (37.3%) and AIDS care unit (24.0%) guaranteed PPE. More interestingly, personnel such as those working at Emergency and internal Medicine who are regularly in contact with suspected cases or known ill patients only represented 8.7% and 0.7% with permanent PPE available, respectively.

Due to shortage, face masks were replaced with tissues in-house made face masks. The inadequate availability of PPE may have contributed significantly to the additional risk of COVID-19 infection amongst HP as already reported by Worby et al. [37] and Zhang et al. [38]. Moreover, regarding building capacity on IPC, the burden of COVID-19 weakened the established infection prevention and control across many health settings as reported by Mhango et al. [39]. Despite the high observed rate of the IPC of HPs towards COVID-19 containment compared to other communities, the overloaded working environment with the shortage of PPE made HP more vulnerable to COVID-19 infection [40, 41]. Additionally, most of the training took place online, in order to respect barrier measures. Training were therefore weakened in the context of network issues.

All those working at the central level had PPE. In contrast only 20% was recorded at the peripheral level. This could be explained by the fact that those at the district work at the operational level in the health pyramid. If the follow up is not well done, they can be easily forgotten when PPE are available, meanwhile those at the central level, as policy makers, will ensure first to be protected to better protect others. The rapidly evolving situation of the epidemic during the second wave required quick actions such as making face mask-wearing and social distancing amongst others compulsory [42]. Following this, it was shown that not using this PPE increased the probability of a positive SARS-CoV-2 antibody test in HP [39]. Moreover Worby et al. [37] demonstrated that face masks can reduce total infections delay the peak time of the epidemic. It was therefore surprising to observe here that those wearing face masks regularly were significantly antibody seropositive (OR 1.86, CI: 1.23–2.8, p = 0.003). The possible reasons might be that face masks were not correctly used as recommended by the WHO guidance [43]. This inability to be consistent was also reported recently by Cho et al. [44]. Therefore, it is not enough to provide PPE, there is need to ensure they are properly used and this could be done by training. In our study only ¼ of HP had attended training on IPC and the difference was significant between HP from Public and private health facilities (P<0.05). This can be attributed to the national task force for COVID-19, as priority was given to public servants first to be trained for the government to efficiently respond to the outbreak.

## Conclusion

Health care facilities still faced enormous challenges to contain the spread of SARS-CoV-2 during the second wave of COVID-19 pandemic in Cameroon. Active infection amongst health professionals were within the range of pandemic control suggesting a good response of the health system in terms of protecting those at the frontlines. However, around two-fifths of HP have had contact with the virus, indicating that they remain a population at risk of COVID-19 and other similarly-transmitted epidemic prone diseases. Given that Anti-SARS-CoV-2 antibodies were higher in active infections, there is need of vaccine to achieve protectiveness; and optimal response also requires capacity building of health personnel laying emphasis on those in the private sector and those working at the peripheral level of the health pyramid. Production of PPE in Cameroon should be promoted to avoid shortage as these are key requirements in the process of mitigation of the spread of the virus. Such actions would contribute in strengthening the health system response when challenged by a future pandemic or epidemic.

## Supporting information

S1 Text (XLSX)
